# Community Readiness Assessment of the “Take TIME for Your Child’s Health” Intervention

**DOI:** 10.3390/healthcare11172386

**Published:** 2023-08-24

**Authors:** Lisa He, Ingrid Svelnis, Amanda Ferraro, Brian W. McCrindle, Tyler Moon, Art Salmon, Patricia E. Longmuir

**Affiliations:** 1 Faculty of Medicine, University of Ottawa, Ottawa, ON K1H 8M5, Canada; lhe037@uottawa.ca; 2 Township of Uxbridge, Uxbridge, ON L9P 1H1, Canada; ingrid.svelnis@brock.ca (I.S.); aferraro@uxbridge.ca (A.F.); 3 Division of Cardiology, The Hospital for Sick Children, Toronto, ON M5G 1X8, Canada; brian.mccrindle@sickkids.ca; 4 Heart and Stroke Foundation, Toronto, ON M4P 1E4, Canada; tylermoon1979@gmail.com; 5 Canadian Fitness and Lifestyle Research, Ottawa, ON K1B 0A9, Canada; drfish@sympatico.ca; 6 Children’s Hospital of Eastern Ontario Research Institute, Ottawa, ON K1H 8L1, Canada

**Keywords:** preschool, young children, health promotion, community intervention, physical activity, healthy eating, injury prevention, smoking avoidance

## Abstract

Take TIME (Tobacco-free, Injury-free, Moving daily, Eating healthy) was an early intervention strategy targeting community readiness to support healthy lifestyles for young children in Uxbridge, Canada. This study aimed to assess the effectiveness of Take TIME using the Community Readiness Model adapted for childhood obesity prevention. Six interviews were completed in Uxbridge, before and after the intervention, with purposively selected community leaders in education, political, business, religious, not-for-profit, and healthcare fields. Each interview was rated independently by two scorers. Interview content was scored (scale from 1 to 9, with 1 being no awareness and 9 being a high level of community ownership) according to the Community Readiness Model criteria on six dimensions, with overall readiness calculated as the mean score of all dimensions. T-tests compared readiness by time-point and between communities. Overall community readiness significantly improved (*p* = 0.03) in Uxbridge from pre-intervention (3.63 ± 1.14 vague awareness) to post-intervention (5.21 ± 0.97 preparation). Seven interviews were also completed with leaders in the matched town of Rockwood, Canada which served as the control community. Rockwood readiness was close to the Uxbridge post-intervention score (5.35 ± 1.11). Results indicated increased awareness and leadership support post-intervention in Uxbridge, but further improvements in community knowledge, formalized efforts, and additional leadership support are desired. Take TIME increased community readiness to support healthy lifestyles for young children and may be useful to other communities at similar stages, given its theoretical alignment with the community readiness model. Future research should investigate the impact of Take TIME in demographically diverse communities and appropriate interventions to move communities from the preparation to the action stage.

## 1. Introduction

Children are engaging in less physical activity and worse dietary habits, resulting in an increased risk of detrimental health effects such as childhood obesity, diabetes, hypertension, heart disease, and other conditions. Physical activity declines from age 3, meaning that children are subject to many more years of unhealthy lifestyle behaviors [1]. Interventions are vital to change this trajectory, but communities have different needs and respond variably to interventions. The Community Readiness Model, developed by the Tri-Ethnic Center for Prevention Research at Colorado State University, integrates a community’s culture, resources, and level of readiness to develop stage-appropriate strategies [2]. The model considers six dimensions to classify a community’s preparedness to combat an issue. Interventions matched to community readiness are vital; they are challenging enough to create change without being too ambitious [2]. Studying community readiness is important because of the implications for public health and community intervention planning. Findholt [3] reported the model was effective in gauging community readiness for addressing childhood obesity. 

A scoping review conducted in 2022 found 17 studies worldwide that used the community readiness model for childhood obesity prevention. The average community readiness scores from studies without an intervention ranged from 1.6 to 6.08 [4]. These studies demonstrate the effective use of the community readiness model for assessing community preparedness to prevent childhood obesity. The review also identified five studies comparing the results of community readiness before and after an intervention [4]. Readiness scores increased after an intervention period of 2 to 5 years following “It’s Your Move!” in Australian schools (score change 2.40 to 4.80) [5], “Obesity Prevention and Lifestyle” in South Australia (leadership dimension scores 3.51 to 5.23) [6] “SaludABLEOmaha” in a Midwestern USA Latino community (scores 3.00 to 5.00) [7], and “Childhood Obesity Prevention Program” in Georgia, USA (scores 4.77 to 6.47) [8]. Only “Ready for Recess” in the Midwestern USA did not impact community readiness (scores 3.26 to 3.16) [9]. Intervention success was attributed to providing financial support and technical assistance as part of a collaborative grant strategy [8], social marketing and social media techniques to shift community knowledge, values, and norms around obesity [7], and increasing a community’s capacity to identify, mobilize, and address public health problems by the development of knowledge, skills, structures, and resources [5]. Schroder and colleagues suggested the need to investigate whether there is a minimum threshold for intervention duration before an increase in readiness can be seen [4]. Furthermore, previous studies were conducted in the United States and Australia, leaving a gap in knowledge for Canadian communities. 

The primary objective of this study was to evaluate the effectiveness of the Take TIME intervention using the community readiness model. The Take TIME intervention, conducted over a period of 7 months, targeted parents and caregivers of children up to eight years old and encompassed physical activity and healthy eating events, information dissemination, and guidelines for community organizations in Uxbridge, Canada (intervention site). Take TIME was based on needs determined through consultations at recreation, library, and retail facilities which indicated that organizations were open to enhancing policies and programs to improve children’s health, policy changes and education were needed, coordination between groups would enhance program availability, and better use of existing facilities was needed. Community readiness was evaluated before and after the intervention. The community readiness model was selected for this purpose because it is an efficient, inexpensive, and easy-to-use tool that has been widely accepted and utilized, which lends credence to its validity [2]. Just as there are recognized stages for individual behavior change that require different intervention approaches, understanding the stage of community readiness for change is important to guide the design of effective interventions. Interventions aligned with community readiness foster greater community development (as communities move to higher levels of readiness), enabling the introduction of broader public health initiatives focused on actions and programs. It was hypothesized that Take TIME would increase community readiness to support healthier lifestyles for young children. As such, it would provide a model and framework for community initiatives in other locations. A secondary objective was to compare community readiness in Uxbridge to Rockwood, a matched control community. Healthy lifestyle choices were hypothesized to be similar in the intervention and control communities prior to Take TIME, but more prevalent in Uxbridge post-intervention. 

## 2. Materials and Methods

### 2.1. Overview

We conducted a prospective pre-post-intervention trial to evaluate the effectiveness of the Take TIME intervention in comparison to a control community. Initial interviews established the readiness of the community to support healthy lifestyles among young children before the Take TIME intervention was introduced. Interviews after the Take TIME intervention were compared to the initial interviews to assess the change in community readiness. Interviews in a control community that did not receive the intervention were used as a comparison. This study was approved by the Research Ethics Board at the Hospital for Sick Children (file number 1000016564). 

### 2.2. Intervention

Take TIME occurred from October 2010 to May 2011 in Uxbridge, Ontario. The four target health behaviors were daily physical activity, healthy eating, reducing injuries, and reducing tobacco exposure. The intervention involved both community events and information dissemination/education/knowledge sharing. 

At least two, free, 1–3 h events open to all community members and using community facilities were hosted each month by community organizations. Families could learn about physical activity opportunities that they could enjoy in the community by participating in the events. Each event also included an educational component about healthy eating, injury prevention, or reducing tobacco exposure. A complete list of the community events is provided in the Supplementary File S1. 

Information about Take TIME was disseminated through community publications/notice boards, schools, a website (linked to the Township of Uxbridge website), and local media. Knowledge sharing about healthy lifestyles was accomplished through parent information sessions at schools and clubs. A garden-sharing project paired older adults living in their own homes with young families wishing to grow their own food in order to facilitate awareness of healthy eating. The Take TIME website also provided two daily tips describing changes that could be implemented in 1 or 5 min. Sponsors provided materials, equipment, and healthy snacks at each event. Resources to plan a Take TIME initiative are available at www.taketimeontario.ca.

### 2.3. Assessment Procedures

Our primary outcome was community readiness based on the model developed in 1994 by the Tri-Ethnic Center for Prevention Research at Colorado State University [2] and adapted for childhood obesity prevention by Findholt [3]. Take TIME effectiveness was evaluated through interviews with community leaders according to Community Readiness Model procedures [3]. Uxbridge pre-intervention interviews were completed in September 2010; post-campaign interviews were completed in June 2011. During each evaluation timeframe, six key community leaders were sought to represent political, religious, educational, not-for-profit, private, and community health sectors. Four to six interviews are sufficient with accuracy further improved by restricting respondents to people who have been in the community for over one year [2]. Increasing the number of key informants to 12–15 does not change community readiness scores or readiness stage [10]. Respondents who have different community roles with different community connections are targeted in order to obtain unique perspectives and an accurate reflection of what is happening in the community [2]. As such, three leaders were identified within each category and two willing to participate were randomly assigned to the pre- or post-intervention evaluation. Leaders completing pre-campaign interviews were ineligible for post-campaign interviews. If leaders were unavailable/declined, an alternate was chosen from the initial list. Rockwood, Canada was matched as the control community based on size, household composition, population density, distance from Toronto, and economic status. Interviews with randomly selected Rockwood leaders were completed November 2010 to May 2011. Questions followed the established interview guide to maintain consistency amongst respondents and follow-up questions were used to clarify responses if necessary. The interviews were digitally audio-recorded, edited to de-identify the files, and transcribed for analysis.

### 2.4. Scoring and Statistical Analyses

Each interview was independently assessed by two raters using the criterion-anchored rating scales for the six community readiness dimensions [2]: A-community efforts; B-community knowledge of efforts; C-extent of community leader support; D-community climate/prevailing attitude; E-community knowledge of issue; F-resources (people, time, money, space, etc.) to support efforts [3]. The average score among all 6 interviews was calculated for each dimension and then dimension scores were averaged to obtain the overall community readiness score. Overall scores were classified according to the stages outlined in the Community Readiness Handbook (Table 1). T-tests were used to make comparisons between the overall readiness score and between each dimension (A–F) for: Rockwood and Uxbridge pre-intervention, Uxbridge pre-intervention, Uxbridge post-intervention, and Rockwood and Uxbridge post-intervention. Cohen’s d was calculated as an indication of a small (d > 0.2), medium (d > 0.5) or large (d > 0.8) effect [11].

### 2.5. Qualitative Analysis

Throughout the process of multiple reviews of each interview by two raters, it was noted that some important concepts, perceptions, and ideas either were not well-represented by the quantitative scoring criteria or would provide greater depth of information for the assigned scores. Therefore, a secondary qualitative analysis was completed to separately identify common themes identified in multiple interviews or differences between the control, pre-intervention, and post-intervention interviews. Themes were identified as statements/ideas evident in at least two interviews per group. Further readings identified specific quotes for each theme. Themes were then compared between interview groups. Finally, quotes that gave novel insights on community climate, community foci, or unique statements/perspectives not otherwise mentioned were identified. An in-depth qualitative analysis was not completed as the goal was to supplement and provide quotes and examples to illustrate the differences identified through the scoring procedure.

## 3. Results

Study participants are described in Table 2. One person from each category was interviewed in each interview group except Rockwood where no private sector leaders were available. An additional not-for-profit leader and community health leader participated.

### 3.1. Community Readiness Scores

**Figure 1:**
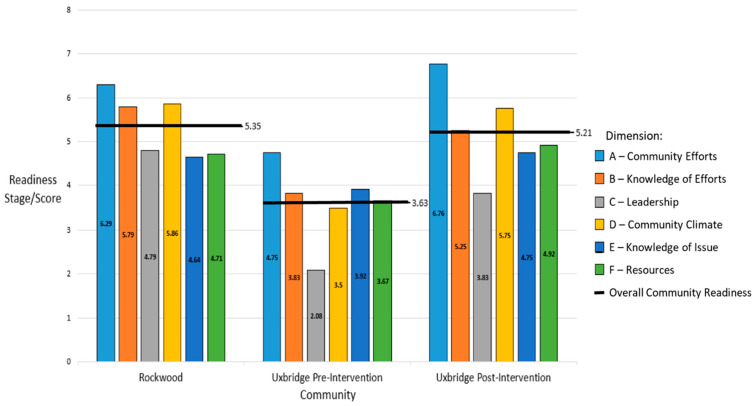
Average Community Readiness Total and Domain Scores by Study Group. The Average Community Readiness Scores [3] displayed including both the individual dimension scores (A through F) along with the Overall Community Readiness Score for each community which is derived from the average of the individual dimension scores.

Community readiness scores for each dimension and overall are displayed in Figure 1. Individual interview scores are displayed in Table 3a, Table 3b, and Table 3c respectively. The control community (Rockwood) was at Stage 5 (Preparation), where some planning on the issue was taking place and there was some support from the community and leadership. The strongest dimensions in Rockwood were for the initiation of community efforts (dimension A, stage 6), community knowledge of efforts (dimension B, stage 5), and community climate (dimension D, stage 5).

Baseline overall readiness in the intervention community (Table 3b) was assessed as vague awareness (Stage 3). This stage is characterized by some community knowledge/concern but no motivation to take action. The strongest dimension in Uxbridge pre-intervention was community efforts (dimension A, stage 4), while the weakest dimension was leadership support (dimension C, stage 2).

Overall readiness increased after the Take TIME intervention, moving the Uxbridge community into the preparation stage (stage 5, Table 3c). The strongest dimension remained community efforts (dimension A, stage 6). Other strong dimensions were community knowledge of efforts (dimension B, stage 5) and community climate (dimension D, stage 5). Leadership support for the issue remained weak (dimension C, stage 3).

### 3.2. Rockwood and Uxbridge Pre-Intervention Comparison

At baseline, support for healthy lifestyles in young children in Rockwood (control community) was significantly higher than in Uxbridge (intervention community, t = 2.74, df = 11, *p* = 0.02, Cohen’s d = 1.53). Community knowledge of efforts (t = 2.34, df = 11, *p* = 0.04, Cohen’s d = 1.29), leadership support (t = 3.23, df = 10, *p* = 0.01, Cohen’s d = 1.78), and community climate (t = 3.31, df = 11, *p* = 0.01, Cohen’s d = 1.83) were higher in Rockwood. The two communities did not differ in efforts to support healthy lifestyles (t = 1.47, df = 10, *p* = 0.17, Cohen’s d = 0.83), community knowledge (t = 1.13, df = 8, *p* = 0.29, Cohen’s d = 0.63) or available resources (t = 1.50, df = 8, *p* = 0.17, Cohen’s d = 0.84).

Qualitative analyses also found more support for healthy lifestyles among young children in Rockwood than Uxbridge pre-intervention. “Rockwood is very interested in health and is health-oriented because we are a veteran community” (health leader). “Living in Rockwood we are fortunate because it is a rural farm community so there is a fair awareness of healthiness to start so a lot of kids are already active” (not-for-profit leader). “I just think we have a young knowledgeable society and realize they have the ability to [change childhood obesity] … it’s a very young community so good parent involvement; never any shortage of coaches” (health leader). Some felt the community did not prioritize the issue enough. “I think people believe we have the power to change but at this point, the community doesn’t feel it is an issue here” (not-for-profit leader Rockwood). The community and local businesses “would do it if asked but have no specific passion or any one person/group stating let’s battle this issue!” (Not-for-profit leader). Despite these few opposing views, the overall community climate was supportive of childhood healthy lifestyles. Increased awareness and knowledge was one possible explanatory reason. An education leader explained the “food and beverage policy—speaks about how many children [are] overweight and how this generation is expected to have [a] shorter lifespan than their parents”. A health leader said the “library [is] excellent, they run little seminars—but we do need to educate people more about childhood obesity and greater physical activity—but don’t think Rockwood is as bad as other places”.

Uxbridge had a less cohesive community climate regarding healthy lifestyles for young children. “I know of a health and wellness profession that’s starting, quite a few of them are into health and nutrition, like coaching… the population is well educated” (business leader). “The Uxbridge population is very aware and everyone pulls together to fix things… this is a very caring community. If we had a problem, everyone would come to the front to help fight it” (not-for-profit leader). However, when asked if the community believes they have a responsibility to address the issue, one leader responded, “that attitude is not prevalent… residents are not campaigning” (religious leader). Others explained that “it’s not that they’re not aware, the information is out there, but if they cannot relate to it then they ignore it” (business leader). “I think there is a general feeling that we should be concerned about it. Getting people to actually do something about it is the issue. Getting people motivated… being concerned about and doing something about it are two different things” (political leader). “There are always kids that opt-out; not everyone buys in… people can be very defensive about lifestyle choices” (educational leader). “I think in general most people just live their lives the way they live them. They’re too busy to think about the next thing”. (health leader). Uxbridge interviews also reflected differing opinions on leadership support. “I think if groups were to come forward and say we have an idea, there would be a positive feeling” (political leader). Others felt “childhood obesity is about a 1 in terms of concern to the leadership in this community” (religious leader). Recognition of efforts also differed. An educational leader said “we have everything: soccer, rugby, hockey, swimming, all kinds of community-wide events, beautiful trails… as for food: the school cafeteria is a teaching cafeteria…there are two classes of food students in there preparing food each day, all based on healthy food choices, no fried food”. Other interviewees claimed that the issue was not discussed much and that efforts did not really exist. “I don’t think they’d do anything about it. You see these things going on and you don’t see anyone voicing a concern … Not paying attention, that’s the problem, no one’s paying attention. However, there are not that many children who are obese” (not-for-profit leader).

### 3.3. Uxbridge Pre- and Post-Intervention Comparison

Community readiness was higher after the Take TIME intervention (t = 2.59, df = 10, *p* = 0.03, Cohen’s d = 1.49). Community climate significantly improved (t = 2.80, df = 9, *p* = 0.02, Cohen’s d = 1.61). Scores for community efforts (t = 2.32, df = 6, *p* = 0.06, Cohen’s d = 1.34), community knowledge of efforts (t = 2.14, df = 10, *p* = 0.06, Cohen’s d = 1.24), leadership support (t = 2.09, df = 9, *p* = 0.07, Cohen’s d = 1.21), community knowledge of the issue (t = 1.16, df = 9, *p* = 0.28, Cohen’s d = 0.67), and resources to support the issue (t = 1.61, df = 10, *p* = 0.14, Cohen’s d = 0.93) increased, but not significantly. After the intervention, only one interview reflected a negative view of the community’s attitude. “I don’t think there is much of a feeling of ownership for that particular issue” (education leader). The remaining interviews were positive. “We are a very active community and parents (at least that I know) are very observant of what children are eating” (political leader). “The community is trying to be active and participate in other activities” (not-for-profit leader). “The general population in this community are fairly well educated and fairly knowledgeable” (health leader). “I think there is a lot the community can do. We have the right tools and people are aware obesity is an important issue” (religious leader). “Everybody is really supportive of the kids up here. We are all very aware that activity is extremely important … we for sure all support the sports so the kids are busy and they are moving around … we certainly wouldn’t want to see obese children” (business leader).

Pre-intervention, healthy lifestyle efforts targeting young children were viewed as “Inaccessible for the lower socioeconomic status group” (religious leader), a view shared by political and education leaders. “The population that … don’t have access to a lot of money, don’t put their kids into activities, have poor diets. The other half of the population seems to be quite healthy, for them the kids are not walking” (health leader).

Although Take TIME aimed to improve accessibility, it remained a concern to many post-intervention. “I think it is probably a smaller group of people who may not have access to the programs whether it is transportation or funds … certain programs are providing the tools for healthier lifestyles but I don’t think parents are necessarily using these” (religious leader). “Not enough is being done to promote the financial relief that is available” (education leader). “Although we have tried to put financial help in place, it is the people that need it the most who are unlikely to ask for it so we cannot reach the help out to them” (political leader). One participant explained that inaccessibility issues are being addressed through “the JUMP start program from Canadian Tire” (business leader).

### 3.4. Rockwood and Uxbridge Post-Intervention Comparison

Post-intervention there was no significant difference in overall community readiness (t = 0.24, df = 11, *p* = 0.82, Cohen’s d = 0.13) or any dimension (community efforts (t = 0.65, df = 8, *p* = 0.53, Cohen’s d = 0.35), community knowledge of efforts (t = 0.67, df = 10, *p* = 0.52, Cohen’s d = 0.37), leadership support (t = 0.98, df = 11, *p* = 0.35, Cohen’s d = 0.55), community climate (t = 0.12, df = 10, *p* = 0.90, Cohen’s d = 0.9), community knowledge of childhood obesity (t = 0.20, df = 9, *p* = 0.85, Cohen’s d = 0.12), resources available (t = 0.33, df = 10, *p* = 0.75, Cohen’s d = 0.19)) between the control and intervention communities. There also seemed to be more general knowledge and awareness of healthy lifestyles for young children after the intervention, although multiple respondents claimed that specific knowledge was lacking. “I think we are aware of general concepts and some of the increased risks like diabetes” (not-for-profit leader). “I think most people know the basics but I don’t know how much detail most people know about it” (religious leader). Leaders also suggested areas needing further improvement. “I don’t think that as a community that we would think we should tolerate the issue for children in terms of weight and inactivity. I just know the community well enough to say that there is a pretty conscious effort to support good eating. There is just nothing that is formalized … I think there has been support and the hospital in the community and all the activities really show that there is good support in the community. I think it just needs to be more formalized” (health leader). “I think leaders offer help, but I think there have to have more defined programs so that the leaders of the community can participate and help fund” (religious leader). “I think that it is a desirable goal to not have any more childhood obesity, but efforts to promote it further are something that goes back to the community leaders” (education leader). A political leader explained that “from a political stand I am aware we help fund some of the sports available in the community … [but] if we leave it up to the leadership to come up with an effort or program it will not happen. If there is something planned I think both businesses and township would support it”.

### 3.5. Emphasizing Physical Activity More Than Healthy Eating

In all evaluations, there were many efforts to support children’s physical activity but fewer related to nutrition. There are “lots of children’s programs… school, scouts, girl guides, baseball and things like that” but when asked to rate healthy food choice efforts the response was “probably about a 5 (out of 10); lower because it seems to me that people with obesity don’t think of it as being a health issue” (religious leader). Other leaders mentioned that it is a “fairly active community…not one activity opportunity that’s missing… sports, soccer, dance, lots of free things,” but when asked about eating specifically said “maybe no effort but lots of awareness that I know of”. A health leader also said there is “nothing organized presently that addresses just childhood obesity… [but] lots of organized sports within the community”. Uxbridge leaders mentioned that Uxbridge has nice trails for exercising. “Our town calls itself the trail capital of Canada”, but when asked about efforts for healthful food choices they said, “I’m not sure about that”.

Leaders also mentioned that healthy eating was more privatized and nutrition more individualized. “I think that the unfortunate part is that because it is done by referral the families are accessing the information through their family physician so it is not a very strong message to the community or not a lot of information for the community as it is done on an individual basis” (health leader). “We have a signage system that people tend to check out. For example at the arena it is very easy for people to check when the season begins for various sports but there isn’t anything comparable for healthy eating”. (education leader).

### 3.6. Healthcare and Schools as Information Sources

Doctor’s offices and family health teams were recognized as places where people could obtain help and information. “We do have a new family health team that have begun to do workshops and I wouldn’t be surprised if exercise or nutrition is a huge part of that” (not-for-profit leader). There was also mention of stigma. “I think there is a stigma around it so going to see a family practitioner is better than going to the gym where people might point out the “fat” kid and make them feel bad” (education leader). Schools were also mentioned by leaders as providing childhood obesity programs, education, and funding in some instances. One participant mentioned, “school and nursery school programs that are very nutritious conscious” (health leader).

### 3.7. Lack of Local Data

The lack of local data was a common and important issue. Many interviewees were unsure whether local data existed and where to access it. “I haven’t seen any. That may come through [the] medical system and I don’t access that” (religious leader). “Never looked for it… wouldn’t know where to start; possibly Stats Canada? … not sure” (education leader). “Haven’t seen it in a hospital or doctors’ offices” (not-for-profit leader). “I don’t know, you would have to [be] looking. There is definitely nothing that springs for me” (education leader).

## 4. Discussion

### 4.1. Key Results

Our study objectives were to evaluate the effectiveness of the Take TIME intervention using the community readiness model and to compare community readiness in Uxbridge to a control community, Rockwood. Study results support our hypothesis that the Take TIME campaign increased the readiness of the Uxbridge community to support healthy lifestyles for young children. Major pre-intervention barriers included attitude towards the issue and inaccessibility of existing efforts. The Take TIME intervention targeted those barriers by increasing public awareness and hosting free events. Post-intervention, improvements were reported in community readiness. Further improvements in community knowledge of the issue, leadership support, and more formalized efforts were desired.

Comparisons between the intervention and matched control communities differed from the study hypothesis. Observed differences at baseline suggest that factors other than demographics were important contributors. The Rockwood Township website emphasizes community participation and volunteerism [12]. This was also evident in the interviews. Respondents claimed that there are “lots of parent volunteers” (education leader) and “it’s a very young community so good parent involvement; never any shortage of coaches” (health leader). Parent engagement improves participation opportunities, and includes volunteering during physical activity opportunities or providing transportation [13]. Higher levels of childhood physical activity lead to continual parent engagement which further facilitates positive physical activity behaviors [13]. Having more families involved in healthy lifestyle habits contributes to a better community climate. Readiness scores reflected these claims, showing higher community knowledge of efforts, leadership supports, and community climate scores in Rockwood than in Uxbridge pre-intervention. Community efforts, knowledge of the issue, and resources were similar at baseline reflecting the matching of factors such as size and economic status.

### 4.2. Importance of Community Climate

After consultations with community members, non-monetary resources and community climate (the underlying personality of a community) were added to the original community readiness model [14]. A critical review of 13 community and organizational readiness assessment models found climate was an essential feature that can impede or foster change and determine whether a community will accept or reject a prevention intervention [15]. Our study found important differences in community climate between the intervention/control communities and pre/post-intervention. Since a large part of the Take TIME intervention involved information dissemination (both through spreading awareness about specific events and generally about healthy lifestyles for young children), increasing public recognition of the prevention-focused intervention may have enhanced the community climate.

The community readiness handbook suggests that efforts at the vague awareness stage be targeted towards raising awareness that the community can do something [2]. Examples include presenting information at events, posting flyers, publishing newspaper articles, and beginning to initiate events [2]. The Take TIME initiative impacted community climate by raising awareness through the Take TIME website, local publications/noticeboards as well as hosting activities and events for the public. This theoretical alignment suggests that Take TIME may be well suited for other communities at the vague awareness stage. Without the community climate dimension, the measurable impact of the Take TIME intervention on the target community in the vague awareness stage would have been limited.

### 4.3. Addressing Leadership Support

In Uxbridge, leadership support was low initially and, although improved, remained the lowest dimension (vague awareness) after the Take TIME intervention. Other studies have shown that larger increases in the leadership dimension of community readiness for childhood obesity prevention were seen with longer intervention duration and smaller population sizes [6]. Given that Take TIME was a relatively shorter intervention (7 months) compared to other interventions in the literature (ranging from 2–5 years), it is possible that significant changes to the leadership dimension would occur if the intervention were continued. Leadership is a key component for the sustainability of a community-wide intervention [16], particularly for health promotion interventions [6]. Leadership impacts both project feasibility and available resources. Engaging local leaders has been deemed fundamental for health promotion as it encourages ownership of local programs [6]. Variability in the leadership dimension reflects issue prioritization [16]. Leaders have a plethora of issues that vie for their limited time [17]. A critical community crisis may force other problems into the background [18]. As such, leaders require motivation to prioritize healthier lifestyles for young children. In this study, leaders were supportive of efforts led by others. Dedicated individuals to spearhead efforts and strong relationships between leaders and staff are critical. Weak relationships result in low implementation fidelity and weak outcomes [9]. Information about the problem accompanied by potential solutions are more likely to obtain leadership action [17]. Leadership scores that decreased after a school-based intervention have been attributed to the making of a premature commitment without sufficient understanding of the effort required [9]. Educating leaders to make responsible decisions when taking on efforts, enhancing leader-staff relationships, one-on-one meetings with key contacts, or beginning evaluation efforts to monitor progress are approaches thought to enhance leadership interest [2].

### 4.4. A Need for Healthy Eating Interventions

Leaders interviewed for our study consistently identified more efforts promoting childhood physical activity than healthy eating. These results align with those in pre-adolescent girls, where readiness for healthy eating was preparation and physical activity was initiation [19]. Lower healthy eating scores suggest nutrition may be a societal issue requiring targeted efforts. Childhood obesity is the result of a complex interplay between genetics, environment, and community [20]. A 2019 scoping review found that 91% of 285 studies addressed the individual level of determinants but that more evidence was needed to explore the role of community, environmental, and policy-level determinants (addressed in only 42% of studies) [20]. The policy level has been deemed an important factor in health as governments are responsible for providing healthy environments for populations by implementing relevant legislation [20]. Studies have shown that in the USA, counties with stronger nutritional laws regarding school lunches, nutritional educational curriculum requirements, and/or food/beverage advertising regulations have lower childhood obesity prevalence [20]. However, other studies have shown that healthy eating initiatives outside of schools are limited and can be more contentious due to school—parent disagreements regarding healthy eating policies [19]. Another important factor is health equity, as childhood obesity tends to be more prevalent in socially disadvantaged populations, such as low-income or minority groups [21]. A negative social environment with poverty, unhealthy eating habits, and poor social cohesion was found to be associated with childhood obesity [21]. As such, the social determinants of health must be targeted to address the societal issue of nutrition and childhood obesity as a whole.

### 4.5. Strengths and Limitations

This novel study utilized the Community Readiness Model to assess target communities and evaluate Take TIME intervention effectiveness. Broad leadership input was obtained. Numerical scores were enhanced with qualitative descriptive results. Scoring was completed separately by two individuals, with consensus through discussion, to reduce bias. Despite these efforts, there were still some limitations in the methodology, data collection, and interpretation of results. One potential limitation is the small sample size given that only two communities were evaluated, only one community received the intervention, and only 6–7 interviews were completed for each group. Although the community readiness handbook states that 6 respondents are often sufficient [2], other studies have claimed that the ideal number may be 10 informants, as that was the point at which community readiness scores showed little variation [10]. Furthermore, there may have been biases in participant selection—both in the families reached by the intervention and in the recruitment of community leaders for interviews. We aimed to reduce this selection bias through the randomization of community leaders and through choosing different leaders for the pre-intervention and post-intervention groups. During the scoring portion of the study, some scores were difficult to assign because interviewees were unsure/unclear of their answers making it difficult to assign a stage of readiness. Questions about whether sports activities alone should count as healthy lifestyle efforts were also raised. Many interviewees identified sports programmes as efforts to combat childhood obesity, even though such programs are not designed to combat the issue directly. When scoring the interviews for this study, higher scores were assigned if communities fulfilled both physical activity and nutrition components. Obesity is a multifaceted issue and addressing both healthy eating and physical activity is important to effectively improve children’s health. Finally, another significant limitation was the reporting of data collected many years ago. It is unfortunate that institutional and personal circumstances beyond our control prevented the more timely publication of these results. However, we strongly believe that these results still have relevance and make an important contribution to the field. Healthy lifestyles for young children remain an important issue in our society today, as evidenced by childhood obesity rates. The findings from our research remain applicable and informative for future intervention design.

### 4.6. Implications

Childhood obesity affects communities around the world and early interventions to promote healthy lifestyles are vital. The Take TIME initiative, aligned with theoretical targets for community readiness, successfully moved Uxbridge from stage 3 vague awareness to stage 5 preparation. This suggests that other communities with limited awareness or not yet prepared to combat childhood obesity (i.e., at stage 3 or 4) may also benefit from the Take TIME initiative. As of 2011, Uxbridge township had a population of 20,623 with a population density of 49 persons per square kilometer [22], making it a rural community. 50.1% of the couples in the 2011 census were couples with children under age 25 at home in Uxbridge, compared to the national figure of 46.9% [22]. Uxbridge also had a high total income with 9.8% of the population having an income in the top five percent ($102,300) and 2.1% in the top one percent ($191,100), compared to 5.0% and 1.0% respectively in all of Canada [22]. Only 6.4% of the population in Uxbridge were low-income compared to 13.9% in Ontario [22]. Finally, in 2011, an estimated 3.9% of the Uxbridge population belonged to a visible minority group, compared to Ontario’s 25.9% [22]. These statistics show that Uxbridge is a more affluent rural community and that the results may be generalizable to other similar communities. In contrast, it is hard to predict whether the Take TIME intervention would yield the same results among other demographic groups, including minority populations or lower-income areas. One optimistic hypothesis would be that an intervention such as Take TIME which provides free activities and awareness to the public could be even more beneficial in a lower-income community in which finances and accessibility may otherwise be even bigger barriers to healthy lifestyles. More research should be carried out to evaluate the impact of an intervention such as Take TIME on other communities or population groups.

Furthermore, given the theoretical alignment of Take TIME to guidelines in the community readiness model, Take TIME may still be useful for other communities at similar stages of readiness irrespective of demographic differences. The community readiness handbook suggests that for the vague awareness stage, the goal is to raise awareness through presenting information at events, posting flyers and posters, conducting surveys and interviews, and publishing articles with more information about the issue and local efforts [2]. For the preplanning stage, the goal is to raise awareness through concrete ideas by investing community leaders in the cause, conducting focus groups, reviewing existing efforts, and increasing media exposure [2]. Schroder’s scoping review summarizes the proposed strategies to increase community readiness in the reviewed studies into four categories: those that increase awareness, those that increase knowledge, those that support communities and connectivity amongst communities, and finally those that promote resources for prevention efforts [4]. The success of the Take TIME intervention may be attributed to the fact that it aligned well with these strategies. Take TIME helped to increase awareness and knowledge about the issue through information dissemination, helped to support the community and encourage collaboration amongst host facilities and organizations for the events, and helped to provide resources and funding to carry out the activities free to the public. Among the 17 studies included in the scoping review of applications of the community readiness model on childhood obesity prevention [4], five studies had a baseline score in stage 4 (preplanning), seven studies had a baseline score in stage 3 (vague awareness), and three studies had a score in either stage 1 (no awareness) or 2 (denial/resistance). The preponderance of communities at the vague awareness or preplanning stages or below suggests that Take TIME would be widely relevant and applicable.

Future work should also evaluate interventions to move a community past the preparation stage. During the preparation stage, planning has begun regarding local problems but often without formally collected data [23]. Communities at this stage may benefit from leadership training, information dissemination, or community empowerment [23]. Local data are essential to convey the problem to the community [24]. Post Take TIME results identified a lack of local data and leadership support; two areas of focus for future interventions. Using surveys to generate accurate local data, gathering local statistics, and holding more diverse and wider-reaching focus groups are recommended to provide the information needed to develop effective strategies [18].

The emphasis on healthcare providers as resources for healthy lifestyles for young children indicates the importance of including this issue within medical education. Physicians need to advocate for healthy childhood lifestyles because they have the power to effect meaningful change. Communities trust physicians and responsibilities come with that trust. Interviewees also identified schools as leaders on this issue, emphasizing the importance of healthy lifestyle lessons in the education system; aligning with the existing literature [17]. Programs that fit into existing school structures, receive teacher support, and require minimal funding or staff time are recommended [17].

## 5. Conclusions

The Take TIME intervention successfully increased community readiness to support efforts to encourage healthier lifestyles for young children, with significant improvements in community climate. Both numerical scores and qualitative results supported our primary hypothesis that community readiness would increase post-Take TIME. The results of this study emphasize the effectiveness of interventions aligned with community readiness for change, and the need for flexible intervention strategies and policies that can be changed to align with changing community readiness. An unexpected finding was that Rockwood was at a higher community readiness level despite being matched on demographics to Uxbridge, suggesting that other factors such as parent engagement and volunteerism may have significant impacts. Studying community readiness is important because of its impacts on public health and intervention development. We believe that the relatively minor impact of community interventions to date may reflect a mismatch between intervention design and community readiness for change. The Take TIME initiative was successful because it targeted the community’s “vague awareness” of the importance of healthy lifestyles for young children. There were some limitations including a small sample size, possible selection biases, and delays in publishing data. It is hard to predict for sure whether the intervention would yield the same results in other demographic groups, including minority or lower-income populations, but given the theoretical alignment with the community readiness model, it is anticipated that the intervention could be applicable to other communities at the vague awareness stage. More research would need to be carried out to confirm or refute these claims. Further research is also recommended to identify interventions that effectively move communities past the preparation stage. The results of this study emphasize the positive impact of interventions designed to align with a community’s readiness for change, offering a specific strategy for optimizing the changes observed with intervention initiatives.

## Figures and Tables

**Table 1:** Stages of Community Readiness (Adapted from Plested et al., 2006 [2]).

Score	Name/Stage *	Description
**1**	No Awareness	The issue is not generally recognized by the community or leaders as a problem (or it may truly not be an issue).
**2**	Denial/Resistance	At least some community members recognize that it is a concern, but there is little recognition that it might be occurring locally.
**3**	Vague Awareness	Most feel that there is a local concern, but there is no immediate motivation to do anything about it.
**4**	Preplanning	There is clear recognition that something must be conducted, and there may even be a group addressing it. However, efforts are not focused or detailed.
**5**	Preparation	Active leaders begin planning in earnest. The community offers modest support for efforts.
**6**	Initiation	Enough information is available to justify efforts. Activities are underway.
**7**	Stabilization	Activities are supported by administrators or community decision-makers. Staff are trained and experienced.
**8**	Confirmation/Expansion	Efforts are in place. Community members feel comfortable using services, and they support expansions. Local data are regularly obtained.
**9**	High Level of Community Ownership	Detailed and sophisticated knowledge exists about prevalence, causes, and consequences. Effective evaluation guides new directions. The model is applied to other issues.

* Scores are “rounded down” i.e., any score from 1.0 to 1.99 would be considered stage 1, etc.

**Table 2:** Participant/Interviewee Information.

	Number of Participants (Gender, Position)		
Category	Rockwood	Uxbridge Pre-Intervention	Uxbridge Post-Intervention
Political Leaders	1 (M, Elected Member of Township Council)	1 (M, Former Elected Member of Township Council)	1 (F, Elected Member of Township Council)
Religious Leaders	1 (F, Church Minister)	1 (M, Church Pastor)	1 (F, Church Children Organizer and Teacher)
Educational Leaders	1 (F, School Principal)	1 (F, School Principal)	1 (F, Teacher)
Not-for-profit Leaders	2 (F, Soccer Club VP), (F, Manager of Children’s Services)	1 (F, Hospital Auxiliary)	1 (F, Coach and Child Care Provider)
Private Sector Leaders	0	1 (F, Business Owner)	1 (F, Business Owner)
Community Health Leaders	2 (F, Nurse), (M, Chiropractor)	1 (F, Nurse)	1 (F, Nutrition Dietician Specialist)

**Table 3:** (**a**). Rockwood community raw interview scores and averages. (**b**). Uxbridge Community pre-intervention 2010 raw interview scores and averages (**c**). Uxbridge Community post-intervention 2011 raw interview scores and average.

**(a)**										
**Interview:**	**1**	**2**	**3**	**4**	**5**	**6**	**7**	**Total**	**Dimension Score (Average)**	**Standard Deviation**
A: Community Efforts	5.5	6	8	3	8	7	6.5	44	6.29	1.73
B: Knowledge of Efforts	4	6	7	3	8	5.5	7	40.5	5.79	1.78
C: Leadership Support	2	4	6	5.5	6	7	3	33.5	4.79	1.82
D: Community Climate	4	5	8	5	7	5	7	41	5.86	1.46
E: Knowledge of the Issue	3.5	3.5	5.5	5	5	5	5	32.5	4.64	0.80
F: Resources	4	5	6	4	6	4	4	33	4.71	0.95
Overall Readiness Score (average)	3.83	4.92	6.75	4.25	6.67	5.58	5.42		5.35	1.11
**(b)**										
**Interview:**	**1**	**2**	**3**	**4**	**5**	**6**	**Total**		**Dimension Score (Average)**	**Standard Deviation**
A: Community Efforts	3	7.5	3	6	6	3	28.5		4.75	1.99
B: Knowledge of Efforts	2	5.5	3	4.5	4	4	23		3.83	1.21
C: Leadership Support	1	4	1.5	2.5	1	2.5	12.5		2.08	1.16
D: Community Climate	3	5.5	3	3	4	2.5	21		3.50	1.10
E: Knowledge of the Issue	3.5	5	1.5	5	5	3.5	23.5		3.92	1.39
F: Resources	3.5	4	1	4	5.5	4	22		3.67	1.47
Overall Readiness Score (average)	2.67	5.25	2.17	4.17	4.25	3.25			3.63	1.14
**(c)**										
**Interview:**	**1**	**2**	**3**	**4**	**5**	**6**	**Total**		**Dimension Score (Average)**	**Standard Deviation**
A: Community Efforts	7.5	5.5	7	6.5	7	7	40.5		6.75	0.69
B: Knowledge of Efforts	6	4	7	5	5	4.5	31.5		5.25	1.08
C: Leadership Support	6	3	2.5	3	6	2.5	23		3.83	1.69
D: Community Climate	8	4	4.5	5.5	7.5	5	34.5		5.75	1.64
E: Knowledge of the Issue	5.5	3	4	6	5	5	28.5		4.75	1.08
F: Resources	6	3.5	6	4	6	4	39.5		4.92	1.20
Overall Readiness Score (average)	6.50	3.83	5.17	5.00	6.08	4.67			5.21	0.97

## Data Availability

Study data are available on request to the corresponding author.

## Supplementary Materials

The following supporting information can be downloaded at: https://www.mdpi.com/article/10.3390/healthcare11172386/s1, File S1: Description of Take TIME Community Events.
