# Supplementary material for: Community Readiness Assessment of the “Take TIME for Your Child’s Health” Intervention
Source: Healthcare (Basel). 2023 Aug 24;11(17):2386. doi: 10.3390/healthcare11172386 (PMC10487062; doi:10.3390/healthcare11172386)
Supplement: Supplementary file 1 [file healthcare-11-02386-s001.zip › healthcare-2539514-supplementary.pdf]

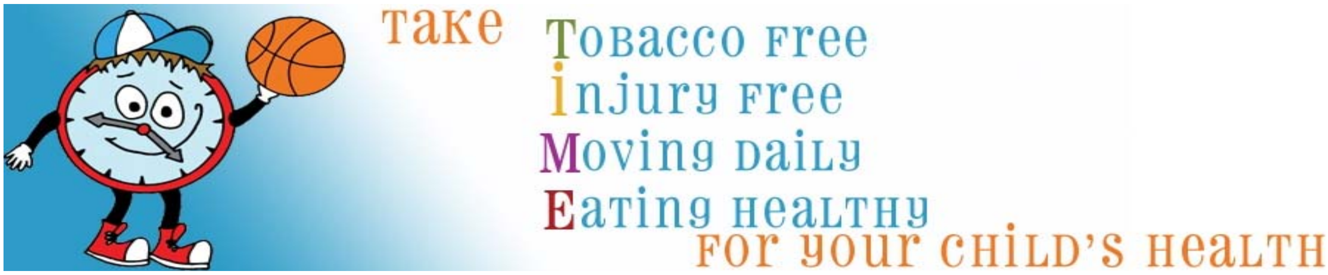

Description of Take TIME Community Events

Event	Date	Time	Location	Hosted by
Baby Boot Camp (parent and baby exercise group)	Thursday Oct 7th to Nov 25th	9:00am-10:00am for babies 1.5-9 mos 10:30am-11:30am for babies 9-18 mos	Uxbridge Baptist Church gym	Sunrise Pregnancy and Family Support Services
Everybody plays soccer	16-Oct-10	1:00pm - 2:00 pm	Uxbridge Public School field (gym if raining)	
Bike ride on the Town Trails	30-Oct-10	10:00am – 10:30 am	Meet at St. Joseph's Catholic School	Uxbridge Cycling Club
Hide and seek	4-Nov-10	10:00am – 10:30am	YMCA Uxbridge	Durham Farm and Rural Family Resources
Hike on the Oak Ridges Trail	13-Nov-10	10:00am – 11:00am	Oak Ridges Trail parking lot on the west side of Conc. 7 (south of Durham 21 and north of Chalk Lake Road)	Oak Ridges Trail Association
Santa Claus parade	27-Nov-10	Check the website for details		Township of Uxbridge
Swim at UxPool	11-Dec-10	3:00pm – 4:00pm	UxPool	Township of Uxbridge
Holiday songs and Festival of Lights	22-Dec-10	6:45pm-7:30pm	Elgin Park Bandshell	Uxbridge Chamber Choir, Bella Nove, Uxbridge BIA
Tim Hortons Free Skating	Xmas break	Check the website for details		Township of Uxbridge
Ice skating	9-Jan-11	1:00pm – 2:00 pm	Arena	Uxbridge Skating Club

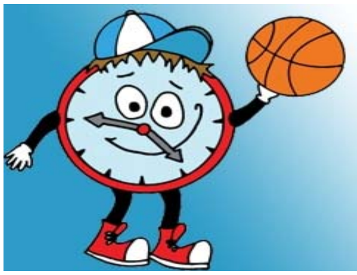

Take **T**obacco free **I**njury free **M**oving daily **E**ating healthy for your child's health

Event	Date	Time	Location	Hosted by
Snow sculptures	22-Jan-11	10:00am – 12:00pm	Siloam Hall	Uxbridge Arts Association
Curling	28-Jan-11	10:00am-12:00pm	Uxbridge Curling Club	Uxbridge Curling Club
Pond hockey	5-Feb-11	10:00am – 11:00am	Elgin Pond	Uxbridge Youth Hockey Association
Family Day	21-Feb-11			
Tobogganing	26-Feb-11	2:00pm – 3:00pm	Hill behind the arena (near baseball diamond)	Uxbridge Youth Centre
Bowling	5-Mar-11	2:00pm-4:00pm	Parish Lanes	Uxbridge Bowling Association
Tim Hortons Free Swimming	March break		Uxbridge Arena	Township of Uxbridge
Bucketball	26-Mar-11	11:00am – 12:30pm	Joseph Gould Public School playground (gym if raining)	Precious Minds
Frisbee golf	16-Apr-11	2:00pm – 4:00pm	Elgin Park	Township of Uxbridge
Geo-cache & scavenger hunt	23-Apr-11	10:00am – 12:00pm	Meet at the Trans Canada Trail kiosk behind the Township office	Uxbridge Boys U-12 Soccer
Huck Finn Day	30-Apr-11		Elgin Pond	Canadian Tire Uxbridge
Bicycle Rodeo	14-May-11	10:00am – 1:00pm	Africycle Parking Lot	CanBike Instructors, Uxbridge Cycling Club, Volunteers of Uxbridge and Roxy Kids
Trail running	28-May-11	11:00am – 11:30am	Countryside Preserve	Uxbridge Optimist Flyers

SickKids

Ontario

HEART & STROKE FOUNDATION OF ONTARIO
Finding answers. For life.
